# Knowledge, Attitude, and Self-Reported Practice Toward Measures for Prevention of the Spread of COVID-19 Among Ugandans: A Nationwide Online Cross-Sectional Survey

**DOI:** 10.3389/fpubh.2020.618731

**Published:** 2020-12-15

**Authors:** Robinson Ssebuufu, Franck Katembo Sikakulya, Simon Binezero Mambo, Lucien Wasingya, Sifa K. Nganza, Bwaga Ibrahim, Patrick Kyamanywa

**Affiliations:** ^1^Faculty of Clinical Medicine and Dentistry, Department of Surgery, Kampala International University Western Campus, Ishaka-Bushenyi, Uganda; ^2^Faculty of Medicine, Université Catholique du Graben, Butembo, Democratic Republic of Congo; ^3^Youth Alliance for Reproductive Health, Goma, Democratic Republic of Congo; ^4^Department of General Surgery, Kitovu Hospital, Masaka, Uganda; ^5^Department of General Surgery, Makerere University, Kampala, Uganda; ^6^Faculty of Clinical Medicine and Dentistry, Department of Obstetrics and Gynecology, Kampala International University Western Campus, Bushenyi, Uganda

**Keywords:** knowledge, attitude, self-reported practice, COVID-19, Ugandan

## Abstract

**Background:** The world is facing the Coronavirus pandemic, which is highly infectious. Several measures have been put in place to prevent its spread among the population. However, for these preventive measures to be effective, the population requires appropriate and sufficient knowledge, attitude, and practices. Thus, a survey to assess knowledge, attitude, and self-reported practice toward measures for prevention of the spread of COVID-19 was conducted among Ugandans.

**Methods:** This was a cross-sectional study conducted among during the lockdown in Uganda. An online structured questionnaire was used, applying a snowballing sampling approach for recruitment of participants 18 years and above and residing in Uganda. Data collection was done from 6th to 15th April 2020, during which 1,763 people participated. We analyzed all data using STATA 14.2, applying appropriate statistical tests.

**Results:** Out of 1,763 participants, 80% were highly knowledgeable. For attitude, 72.4% reported following recommendations given by the Ministry of health to prevent the spread of COVID-19; 89.0% were worried about contracting COVID-19 and 73.3% agreed that COVID-19 can be cured and 99.3% reported good practice toward measures to prevent the spread of COVID-19. According to ordered logistic regression, health workers were 6 times more knowledgeable [aOR:6 (3.51–10.09), *p* < 0.001] followed by teachers [aOR:5.2 (2.6–10.32), *p* < 0.001]; students [aOR:3.2 (1.96–5.33), *p* < 0.001]. On the contrary, the drivers, business entrepreneurs, and security personnel had less knowledge.

**Conclusion:** The results show that the participating Ugandans were knowledgeable and had a positive attitude and good practices. However, there is still a gap in knowledge among drivers, business entrepreneurs, and security personnel. Therefore, there is a need to mobilize the country's population to have the same degree of knowledge, which will have an impact on the attitude and practices toward prevention of the spread of COVID-19.

## Introduction

On December 31, 2019, a respiratory syndrome identified to be caused by a beta-coronavirus was reported in Wuhan, China (1). This syndrome was later officially named as an outbreak of a new coronavirus disease-2019 (COVID-19) by the World Health Organization (WHO) (2), and as severe acute respiratory syndrome coronavirus 2 (SARS-CoV-2) by Coronavirus Study Group (CSG) of the Inter- national Committee on Taxonomy of Viruses, on February 11, 2020 (3, 4).

On March 11, 2020, the WHO declared the COVID-19 as a pandemic due to rapid global spread (5). SARS-CoV-2 presents clinically with fever, dry cough, fatigue, myalgia, and dyspnea (5, 6). The SARS-CoV-2 is transmitted between people through droplets, fomites, and close contact, with possible spread through the eyes, nose, and mouth, but it is not an airborne disease according to the current studies (7). The disease is highly contagious with enormous potential for health, economic, and societal impacts (6).

COVID-19 is rapidly evolving, and currently, there is neither vaccine nor evidence on the effectiveness of potential therapeutic agents (3). As of November 10, 2020, a total of 50,994,215 cases of COVID-19 had been confirmed worldwide (1,892,140 confirmed in Africa), with 1,264,077 deaths (45,605 deaths registered in Africa) giving a case fatality ratio of 3.3% worldwide (2.4% in Africa) (7, 8).

Given the spread of SARS-CoV-2 and its impact on human health, the WHO has recommended strategies to control this pandemic, which include traffic restriction, cancellation of social gatherings, home quarantine, the establishment of clinical care and management strategies, laboratory capacity strengthening, surveillance strategies, case and contact tracing, infection prevention and control, implementation of health measures for travelers, risk communications, and community engagement (8, 9).

Uganda registered her first case of COVID-19 on March 21, 2020, and as of November 10, 2020, according to the Ministry of Health (MOH), the country had registered 14,704 confirmed cases of COVID-19 with 7,836 recoveries and with 133 reported deaths, a case fatality ratio of 0.9% (10). The Uganda government has put measures to contain the spread of the SARS-CoV-2 within the country, including community-based and facility-based measures (10). The key community-based measures include self-isolation for COVID-19 patients and quarantine of contacts and travelers, hand-washing with soap or sanitizers, restriction of movements (lockdown) within and out of the country except for cargo drivers, all gathering places closed such as school, churches, sports, meetings, markets except for food necessities activities and a curfew from 7 pm to 6:30 am, face mask for everyone in the country (10). Facility-based measures have so far included the use of personal protective equipment before handling patients, testing of patients with symptoms, treatment, and contact tracing, and the isolation of the suspected cases and diagnosed cases (10).

Appropriate knowledge, attitudes, and practices could improve the proper uptake of COVID-19 prevention measures. Studies so far done to evaluate the level of KAP toward measures to prevent the spread of COVID-19 among certains African communities and in Uganda in particular have targetted specific groups such as health workers (11), lecturers and students (12) and market vendors (13). These studies show a good knowledge, a positive attitude and good practices among the mentioned participants. However, our study aimed at the general population to determine the variability in knowledge, attitudes, and practices toward measures for prevention of the spread of COVID-19 among different sectors in a bigger Ugandan population. Previous studies on viral disease outbreaks, like SARS in 2003 (14) and Ebola in 2018 (15), have shown that the management and control of an outbreak requires a good understanding by the populations about the disease transmission and prevention to avoid its spread in the community (6).

## Materials and Methods

### Study Design and Setting

This was a nationwide cross-sectional online survey conducted among Ugandans living in any of the four regions (Northern, Central, Eastern, and Western) of the country at the time of the study.

#### Study Population, Sample Size and Sampling Design

All literate Ugandans aged 18 years and above with access to the internet constituted the population of this survey. The population of Uganda stands at 44,269,594, of which 78.4% (34,707,362/44,269,594) are literate (16). In Uganda, 44% (20,000,000/44,269,594) of the general population have a mobile subscription, among whom nearly half are mobile subscribers who are also able to access mobile internet services (17). By June 2018, there were nearly 10 million mobile internet connections in Uganda, a penetration rate of 23% (17).

To calculate the sample size for this study, we hypothesized that at a 99.9% confidence interval (CI), 50% of the respondents would have a satisfactory knowledge level on measures to prevent the spread of COVID-19 in the country. Using the Open Source Epidemiologic Statistics for Public Health (OpenEpi), v.3.01 (Dean AG, Sullivan KM, Soe MM. OpenEpi: www.OpenEpi.com, updated April 6, 2013), the minimum sample size of 1,083 participants was needed, adding a 30% contingency to the sample size, a minimum of 1,408 participants were targeted and at the end of data collection period, a total of 1,768 participants were registered in the study.

Ugandans with a minimal computer literacy level and able to operate a social media account such as an email, WhatsApp, Twitter, or Facebook and consented to participate were included in the survey. Those who had filled in the form but were unable to submit the questionnaire were automatically not reflected and therefore excluded in the survey's database.

### Data Collection and Instrument

Due to the spread of the COVID-19 pandemic and the lockdown policy enforced in the country at the time of data collection, a physical and paper-based questionnaire was not feasible. Data was collected using an online structured questionnaire developed in English using Google forms^1^ with a consent form appended to it.

The questionnaire was developed based on WHO requirements for knowledge, attitudes, and practices (KAP) (18) and from the validated and published study on KAP among Chinese (6) and it was composed of 22 questions focused on several key constructs. The constructs captured by the five questions on socio-demographic characteristics (age, sex, occupation, location, and marital status); eight on knowledge; three on attitude, one on self-reported practice toward the measures put in place to prevent the spread of COVID-19 among Ugandans and one on source of information. The knowledge questions were composed of 12 questions (K1–K12) comprising: (K1) incubation; (K2) mode of transmission of the COVID-19; (K3) clinical presentations of COVID-19; (K4) risk factors for severe illness of COVID-19 and (K5–K12) preventive measures. Three (A1–A3) attitudes questions assessed participant's responses related to their COVID-19 risk perceptions, measures to prevent the spread of the disease and their level of perception about the cure of COVID-19. One (P1) question assessed participants response related to the measures they observed for self-prevention toward COVID-19. Participants were asked their source of information about COVID-19 (Supplementary Table 1).

As the country was under lockdown limiting physical access to potential study participants, social media was used to conduct the survey. The snowball sampling technique was used by asking all initial study participants accessing the online form to recruit their acquaintances fulfilling the eligibility criteria, by sharing the link to the online questionnaire and requesting them to participate within the study timeline. The questionnaire was administered for a period of 10 days from 6th to 15th April 2020. On receiving and clicking the link, the participants were auto-directed to the informed consent page of the survey tool. After reading the preamble and accepting to participate in the study, they were directed to the survey questionnaire.

### Data Analysis and Interpretation

Each rightly mentioned single and multiple choices responses on knowledge questions was scored 1 to give a total score for the knowledge of a particular participant. The range of the knowledge was scored 0–30. The knowledge score was grouped into 3 categories namely: 0–9 (poor), 10–19 (moderate), and 20–30 (high).

Those who “Agreed” or answered “Yes” to the questions related to attitude were scored 1 and those who “Disagreed” or said “No” were coded 0. The category of attitude for this study was a binary variable with score 1 taken to be positive attitude and score 0 as negative attitude.

Each correct response on self-reported practice questions was scored 1 and the incorrect one was scored 0 and then the sum of all the 8 right responses was used to develop a practice score. The self-reported practice was scored as adequate or good practice for those who selected 5–8 correct answers and poor for others who selected 0–4 correct answers.

The raw data was cleaned and entered into Microsoft Excel and exported into STATA 14.2 for processing. Statistical analysis was done using STATA 14.2, where by categorical variables were summarized using frequency tables while continuous variables were summarized using means and standard deviation (SD).

The distances between the categories of the knowledge score were not normally distributed, and therefore, we used the ordered logistic regression for multivariable analysis of knowledge and socio-demographic characteristics indicating adjusted odds ratios (aOR).

The attitude and Self-reported practice and socio-demographic characteristics were analyzed using the Chi-square, *p*-values at univariate analysis, and Odds ratio at 95% CIs. The statistical significance level was set at *p* < 0.05.

### Ethical Considerations

Ethical clearance for the survey was obtained from the Institutional Research Ethical Committee of Kampala International University in Uganda (UG-REC-023/201914). As participants logged in online, a statement regarding the consent to participate in the survey was in the preamble of the questionnaire and could only proceed after reading the consent and accepting to participate in the survey. Participation in this survey was voluntary. Participants were free to withdraw from the survey at any time by not submitting their form online, and there was no repercussion. The participants' identity was concealed as the form does not require any identification. No name or mail was required from the participant. Therefore, the information was obtained and stored anonymously, and this was treated confidentially. Only five members of the research team were allowed to access data, and the principal investigator accessed the entire dataset.

## Results

A total of 1,768 participants completed the online questionnaire. Five (5) participants were excluded from the survey because were aged below 18 years, thus the final sample size considered was 1,763.

### Socio-Demographic Characteristics of Participants

Out of 1,763 participants, 56.9% were male and 50.5% were single. The mean age of the overall respondents was of 32.1(± 9.9) years. 23.7% (418/1,763) were health workers, 14% (247/1,763) farmers and 2.8% (50/1,763) drivers. The majority, 42.9% (756/1,763), of participants were from Western region of Uganda, followed by Central Uganda (29.3%). Other socio-demographic characteristics are shown below in Table 1.

**Table 1:** Socio-demographic characteristics of participants.

**Variable**	**Options**	**Frequency**	**Percent (%)**	**Mean age (SD)**
**Number of participants**		1,763	100	
**Age in complete years**				**32.1 (9.9)**
**Age group in years**	18 to 30	892	50.6	
	31 to 40	549	31.1	
	41 to 50	231	13.1	
	51 and above	91	5.2	
**Sex**	Female	759	43.1	
	Male	1,004	56.9	
**Marital status**	Single	891	50.5	
	Married	811	46.0	
	Divorced	42	2.4	
	Other*	19	1.1	
**Occupation**	Farmers	247	14	
	Business	284	16.1	
	Health workers	418	23.7	
	Household	67	3.8	
	Security	49	2.8	
	Student	346	19.6	
	Teacher	119	6.7	
	Driver	50	2.8	
	Others**	183	10.4	
**Location (region)**	Western	756	42.9	
	Central	517	29.3	
	Eastern	263	14.9	
	Northern	227	12.9	

Other*: Widowed, cohabiting, separated and in relation.

Other**: Technologist and point of entry agent.

### Ordered Logistic Regression of Knowledge Level With Socio-Demographic Characteristics of Participants

The knowledge scores significantly differed across occupation and location (*p* < 0.05) of the study participants but was not significant across age groups, sex and marital status (*p* > 0.05) in ordered logistic regression analysis (Table 2). However, knowledge scores significantly differed across the socio-demographics variables (*p* < 0.05) in the univariate analysis (Supplementary Table 2). The source of information about COVID-19 among participants were as follow: social media 36.8% (648/1,763), television 29.9% (528/1,763), health workers 12.9% (227/1,763), radio 12% (212/1,763), Family and friends 5.2% (91/1,763) and News Papers 3.2% (57/1,763).

**Table 2:** Ordered logistic regression of knowledge level with socio-demographic characteristics of participants.

**Variable**	**Coefficient (95%CI)**	**aOR (95%CI)**	***p*-Value**
**Sample size**	**1,763**		
**Age group in years**			**0.364**
18 to 29	Ref	Ref	
30 to 40	0.2 (−0.2–0.53)	1.2 (0.82–1.7)	0.371
41 to 50	0.1 (−0.38–0.57)	1.1 (0.68–1.76)	0.699
51 and above	−0.3 (−0.92–0.28)	0.7 (0.4–1.32)	0.296
**Sex**			**0.055**
Female	Ref	Ref	
Male	0.3 (−0.01–0.56)	1.3 (0.99–1.75)	0.055
**Marital status**			**0.361**
Single	Ref	Ref	
Married	0.1 (−0.25–0.47)	1.1 (0.78–1.6)	0.542
Divorced	−0.5 (−1.35–0.3)	0.6 (0.26–1.35)	0.213
Others*	−0.5 (−1.81–0.89)	0.6 (0.16–2.43)	0.502
**Occupation**			**0.001**
Farmers	Ref	Ref	
Business	0.3 (−0.1–0.7)	1.3 (0.9–2.01)	0.145
Health workers	1.8 (1.26–2.31)	6 (3.51–10.09)	0.001
Household	0.3 (−0.3–0.98)	1.4 (0.74–2.67)	0.295
Security	0.7 (−0.1–1.44)	2 (0.91–4.21)	0.087
Student	1.2 (0.67–1.67)	3.2 (1.96–5.33)	0.001
Teacher	1.6 (0.96–2.33)	5.2 (2.6–10.32)	0.001
Driver	−0.4 (−1.12–0.22)	0.6 (0.33–1.25)	0.192
Others**	1.9 (1.2–2.6)	6.7 (3.32–13.4)	0.001
**Location (region)**			**0.001**
Western	Ref	Ref	
Central	0.4 (0.03–0.73)	1.5 (1.03–2.08)	0.034
Eastern	1.1 (0.61–1.5)	2.9 (1.84–4.48)	0.001
Northern	0.6 (0.12–1.05)	1.8 (1.12–2.85)	0.014

*aOR, adjusted odds ratio; Ref, reference variable group*.

Other*: Widowed, cohabiting, separated and in relation.

Other**: Technologist and point of entry agent.

Eighty percent (1,411/1,763) of the study participants had high knowledge about COVID-19, 18.3% (323/1,763) moderate knowledge and 1.7% (29/1,763) poor knowledge (Supplementary Table 2).

The ordered logistic regression of knowledge level (Table 2) shows that health workers [aOR:6 (3.51–10.09), *p* < 0.001]; teachers [OR:5.2 (2.6–10.32), *p* < 0.001]; students [aOR:3.2 (1.96–5.33), *p* < 0.001] were significantly associated with a high level of knowledge toward measures to prevent the spread of COVID-19. On contrary, being a business merchant [OR:1.3 (0.9–2.01), *p* : 0.145]; security agent [aOR: 2 (0.91–4.21), *p* : 0.087], household-wife [aOR: 1.4(0.74–2.67), *p* : 0.295] and driver [0.6 (0.33–1.25), *p* : 0.069] were not significantly associated with a high knowledge about COVID-19 which is supported by the prevalence of knowledge in Supplementary Table. There was no statistically significant difference in knowledge on prevention of the spread of COVID-19 among participants regarding their location.

### Attitude Toward Measures to Prevent the Spread of COVID-19 With Socio-Demographic Characteristics of Participants

**Figure 1:**
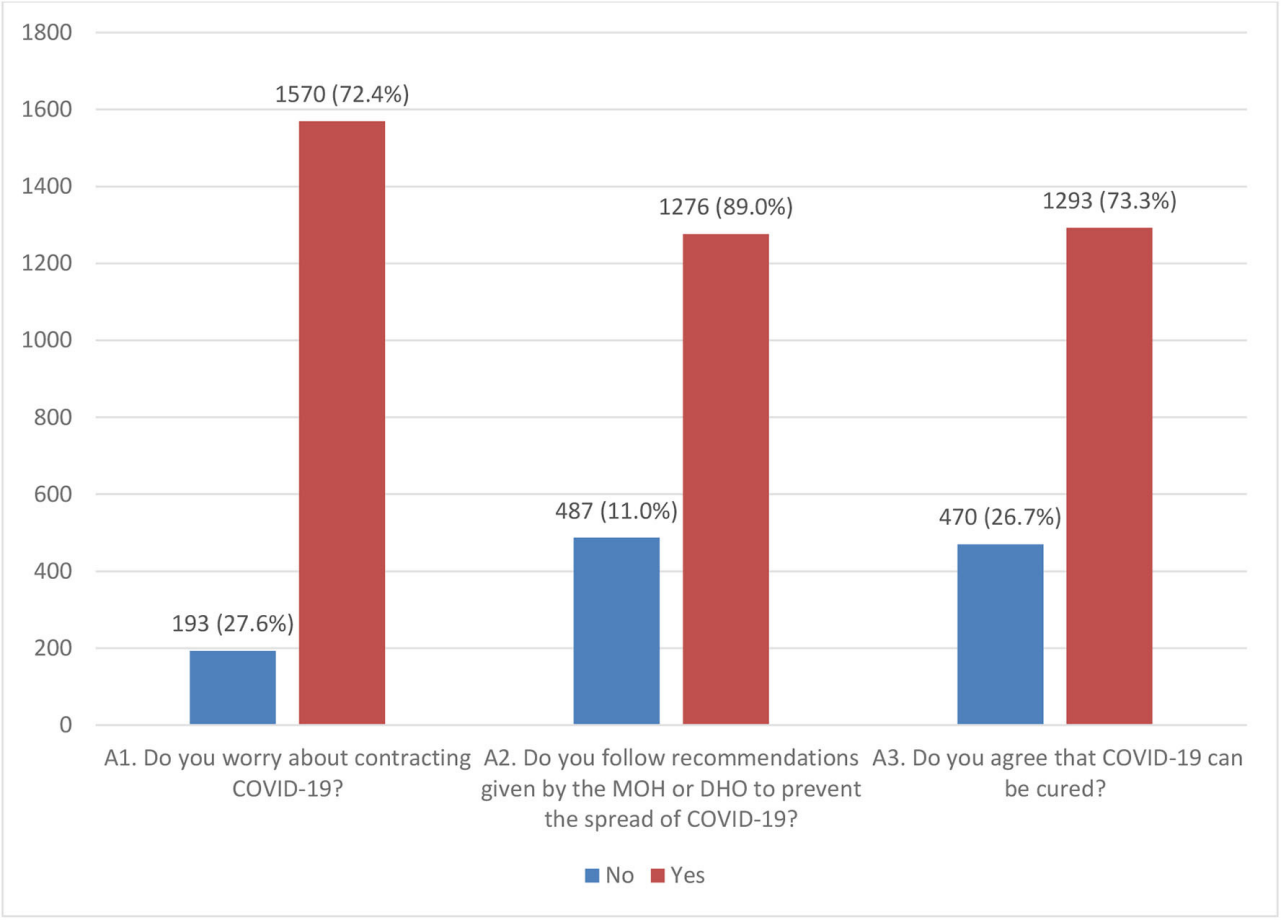
Attitude toward measures to prevent the spread of COVID-19 among Ugandans. MOH, Ministry of Health; DHO, district health officer.

Most participants [72.4% (1,276/1,763)] followed recommendations that have been given by the MOH or DHO to prevent the spread of COVID-19 but 27.6% (487/1,763) did not follow the recommendations; 89.0% (1,570/1,763) were worried about contracting COVID-19 and 73.3% (1,293/1,763) agreed that COVID-19 can be cured (Figure 1). The attitude about contracting COVID-19 (A1) varied across sex, marital status and location (*p* < 0.05). The attitude on following the recommendations (A2) and on agreeing that COVID-19 can be cured (A3) differed across sex and occupation (Table 3). The distribution of high knowledge on measures to prevent the spread of COVID-19 among participants was significant for positive attitude on A1 and A3 but not A2 (Table 3).

**Table 3:** Association of attitude with socio-demographic characteristics of participants.

**Variable**	**All (%)**	**A1. Do you worry about contracting COVID-19?**			**A2. Do you follow recommendations given by the MOH or DHO to prevent the spread of COVID-19?**			**A3. Do you agree that COVID-19 can be cured?**		
		**Positive (%)**	**Negative (%)**	***p***	**Positive (%)**	**Negative (%)**	***p***	**Positive (%)**	**Negative (%)**	***p***
Sample size	1,763 (100)	1,570 (89.1)	193 (10.9)		1,276 (72.4)	487 (27.6)		1,293 (73.3)	470 (26.7)	
Age group in years				0.102			0.703			0.832
18–29	892 (100)	779 (87.3)	113 (12.7)		650 (72.9)	242 (27.1)		658 (73.8)	234 (26.2)	
30–40	549 (100)	495 (90.2)	54 (9.8)		398 (72.5)	151 (27.5)		403 (73.4)	146 (26.6)	
41–50	231 (100)	213 (92.2)	18 (7.8)		167 (72.3)	64 (27.7)		169 (73.2)	62 (26.8)	
51 and above	91 (100)	83 (91.2)	8 (8.8)		61 (67)	30 (33)		63 (69.2)	28 (30.8)	
Sex				0.032			0.009			0.015
Female	759 (100)	662 (87.2)	97 (12.8)		525 (69.2)	234 (30.8)		579 (76.3)	180 (23.7)	
Male	1,004 (100)	908 (90.4)	96 (9.6)		751 (74.8)	253 (25.2)		714 (71.1)	290 (28.9)	
Marital status				0.001			0.219			0.318
Single	891 (100)	778 (87.3)	113 (12.7)		639 (71.7)	252 (28.3)		660 (74.1)	231 (25.9)	
Married	811 (100)	743 (91.6)	68 (8.4)		598 (73.7)	213 (26.3)		594 (73.2)	217 (26.8)	
Divorced	42 (100)	31 (73.8)	11 (26.2)		25 (59.5)	17 (40.5)		28 (66.7)	14 (33.3)	
Others*	19 (100)	18 (94.7)	1 (5.3)		14 (73.7)	5 (26.3)		11 (57.9)	8 (42.1)	
Occupation				0.051			0.001			0.001
Farmers	247 (100)	213 (86.2)	34 (13.8)		125 (50.6)	122 (49.4)		196 (79.4)	51 (20.6)	
Business	284 (100)	262 (92.3)	22 (7.7)		201 (70.8)	83 (29.2)		225 (79.2)	59 (20.8)	
Health workers	418 (100)	385 (92.1)	33 (7.9)		342 (81.8)	76 (18.2)		293 (70.1)	125 (29.9)	
Household	67 (100)	58 (86.6)	9 (13.4)		39 (58.2)	28 (41.8)		52 (77.6)	15 (22.4)	
Security	49 (100)	43 (87.8)	6 (12.2)		36 (73.5)	13 (26.5)		43 (87.8)	6 (12.2)	
Student	346 (100)	295 (85.3)	51 (14.7)		268 (77.5)	78 (22.5)		233 (67.3)	113 (32.7)	
Teacher	119 (100)	104 (87.4)	15 (12.6)		96 (80.7)	23 (19.3)		89 (74.8)	30 (25.2)	
Driver	50 (100)	46 (92)	4 (8)		29 (58)	21 (42)		35 (70)	15 (30)	
Others**	183 (100)	164 (89.6)	19 (10.4)		140 (76.5)	43 (23.5)		127 (69.4)	56 (30.6)	
Location (region)				0.039			0.001			0.386
Western	756 (100)	688 (91)	68 (9)		616 (81.5)	140 (18.5)		552 (73)	204 (27)	
Central	517 (100)	444 (85.9)	73 (14.1)		330 (63.8)	187 (36.2)		386 (74.7)	131 (25.3)	
Eastern	263 (100)	235 (89.4)	28 (10.6)		182 (69.2)	81 (30.8)		198 (75.3)	65 (24.7)	
Northern	227 (100)	203 (89.4)	24 (10.6)		148 (65.2)	79 (34.8)		157 (69.2)	70 (30.8)	
Knowledge				0.001			0.116			0.001
Poorly	29 (100)	5 (17.2)	24 (82.8)		17 (58.6)	12 (41.4)		16 (55.2)	13 (44.8)	
Moderate	323 (100)	167 (51.7)	156 (48.3)		245 (75.9)	78 (24.1)		270 (83.6)	53 (16.4)	
Highly	1,411 (100)	1,104 (78.2)	307 (21.8)		1,031 (73.1)	380 (26.9)		1,284 (91.0)	127 (9.0)	

*aOR, adjusted odds ratio; Ref, reference variable group*.

Other*: Widowed, cohabiting, separated and in relation.

Other**: Technologist and point of entry agent.

### Self-Reported Practice Toward Measures for Prevention of the Spread of COVID-19 With Socio-Demographic Characteristics of Participants

Participants reported good practice of 99.26% for self-monitoring, use of face masks, washing hands, application of social distancing respectively; 85.25% for house cleaning and ventilation; 68.29% of staying at home and avoiding gathering; 30.97% of applying respiratory etiquette. But some of participants reported applying social distancing of <1 m (14.01%) and some (0.7%) did not follow any of the mentioned measures to prevent the spread of COVID-19 in Uganda (Table 4). The practices differed significantly across sex, marital status and occupation of participants (*p* < 0.05). Most participants (99.3%) reported having adequate practice and 0.7% (13/1,763) reported poor practice toward measures for prevention of the spread of COVID-19 (Table 5).

**Table 4:** Reported practices to prevent the spread of COVID-19 among Ugandans.

	**Self-reported practices items (multiple response)**	**Frequency**	**Percent**
1	Self-monitoring	1,750	99.26
2	Use of masks	1,750	99.26
3	Hand washing	1,750	99.26
4	Social distancing of more than two meters	1,750	99.26
5	House cleaning and ventilation	1,503	85.25
6	Avoid gatherings	1,204	68.29
7	Stay at home	1,204	68.29
8	Respiratory etiquette	546	30.97
9	Social distancing of <1 m	247	14.01
10	I don't know	13	0.74

**Table 5:** Self-reported practice toward measures for prevention of the spread of COVID-19 with socio-demographic characteristics.

**Variable**	**Total (%)**	**Good (%)**	**Poor (%)**	***p*-Value**
Sample size	1,763 (100)	1,750 (99.3)	13 (0.7)	
Age group in years				
18 to 29	892 (100)	888 (99.6)	4 (0.4)	0.494
30 to 40	549 (100)	544 (99.1)	5 (0.9)	
41 to 50	231 (100)	228 (98.7)	3 (1.3)	
51 and above	91 (100)	90 (98.9)	1 (1.1)	
Sex				
Female	759 (100)	749 (98.7)	10 (1.3)	0.013
Male	1,004 (100)	1,001 (99.7)	3 (0.3)	
Marital status				
Single	891 (100)	886 (99.4)	5 (0.6)	0.002
Married	811 (100)	806 (99.4)	5 (0.6)	
Divorced	42 (100)	40 (95.2)	2 (4.8)	
Others*	19 (100)	18 (94.7)	1 (5.3)	
Occupation				
Farmers	247 (100)	239 (96.8)	8 (3.2)	0.001
Business	284 (100)	283 (99.6)	1 (0.4)	
Health workers	418 (100)	417 (99.8)	1 (0.2)	
Household	67 (100)	66 (98.5)	1 (1.5)	
Security	49 (100)	49 (100)	0 (0)	
Student	346 (100)	346 (100)	0 (0)	
Teacher	119 (100)	119 (100)	0 (0)	
Driver	50 (100)	49 (98)	1 (2)	
Others**	183 (100)	182 (99.5)	1 (0.5)	
Location (region)				
Western	756 (100)	753 (99.6)	3 (0.4)	0.087
Central	517 (100)	509 (98.5)	8 (1.5)	
Eastern	263 (100)	262 (99.6)	1 (0.4)	
Northern	227 (100)	226 (99.6)	1 (0.4)	

Other*: Widowed, cohabiting, separated and in relation.

Other**: Technologist and point of entry agent.

## Discussion

Currently, the world faces the coronavirus pandemic, which is highly infectious; measures have been put in place to prevent its spread among the population across the world. The population requires an appropriate and sufficient knowledge about these measures, their importance, and how to apply them appropriately (8, 9). When a human population faces an outbreak, changes in behavior in response to the disease can alter the progression of the infectious agent. In particular, people aware of a disease in their proximity can take measures to reduce their susceptibility (18).

However, beyond a critical infection rate, spreading awareness can slow down the spread of the disease and lower the final incidence, but it cannot completely stop it from reaching epidemic proportions and taking over large parts of the population (6) as have been observed in the 2003 outbreak of SARS in Hong Kong (19).

Ten days after the first case of COVID-19 was confirmed in Uganda, we conducted a nationwide online survey on Knowledge, attitudes, and self-reported practice toward measures for prevention of the spread of COVID-19 among the Ugandan population. We found that 80% of participants were highly knowledgeable toward measures for the prevention of the spread of COVID-19 among Ugandans. This result is similar to the knowledge rate (90%) found among Chinese residents during a quick online survey on COVID-19 (6) and during the Ebola outbreaks in Sierra Leone in 2014 (20) and DRC in 2018 [12 = 15] but higher than findings (69%) by Olum et al. (11) among Health Workers in Uganda and by Hager et al. among communities in Nigeria and Egypt (61.6%) (21). Our findings could be explained by the fact that the COVID-19 found Ugandans already familiar with observing similar measures to prevent the spread of some other highly infectious diseases within the country such as Ebola and Marburg disease (22). The ordered logistic regression from our survey showed that the level of knowledge was significantly associated with a certain degree of education level as per health worker, teacher, and student. This survey included all Ugandans with a minimal computer literacy level which is 78.4% of all the Ugandan population (17). The Ministry of Health of Uganda uses social media to post information related to measures for prevention of the spread of the pandemic within the country and this could explain the findings mentioned above which could be different among uneducated people. Zhong et al. findings related to Knowledge in China explain their findings by the fact that most respondents during their survey held an associate's degree or higher (6). The Uganda government could use these categories of participants as a strategy to reach out and sensitize the uneducated population about measures to be observed in the country.

Most participants reported positive attitude on following recommendations that have been provided by the Ministry of Health or directorate of district health officer to prevent the spread of COVID-19. Even if the participants agreed that COVID-19 can be cured (73.3%), most of them were worried of contracting COVID-19 (89.1%). These findings can be explained by the high level of knowledge among participants, and also, the country being under lockdown as one of the measures to prevent the spread of the pandemic. A study conducted about KAP of COVID-19 in the Philippines found that most of participants were afraid of contracting COVID-19 (19). Reuben et al. found an association of good knowledge and positive attitude among participants about COVID-19 (23). In China, a survey revealed that most population took precautions to prevent infection by COVID-19 such as not going to crowded places and wearing masks when going outside but with an optimistic attitude toward COVID-19 which could be attributed to the stringent prevention and control measures implemented by governments such as banning public gatherings (6). In Nigeria, a study evaluating the KAP of Ebola outbreak among secondary school children found an association between poor knowledge and negative attitude toward the outbreak (20). In this survey, householder and driver occupation were associated with a negative attitude. During the Ebola outbreak in DRC and Guinea, it was found that a group of participants had a negative attitude toward measures for prevention of Ebola in their respective areas (15, 24) the same as for a study done among Chinese residents during the coronavirus pandemic (6). Among confirmed cases in Uganda, one-third are truck drivers who are coming from surrounding countries (10). The result gives useful information that the government has to increase sensitization among these categories of people about measures toward prevention of the spread of COVID-19, which can be considered as a cross border infection in Uganda and the East African region. Households can get information from the students, health workers (11, 12) and those who have shown an immense knowledge about measures to be observed and the government can also tap into such categories of people in implementing strategies to control the pandemic within the country.

As of November 10, 2020, Uganda had registered 14,704 confirmed cases of COVID-19 with 7,836 recoveries and with 133 reported deaths, a case fatality ratio of 0.9% (10), and this low case fatality ratio can be explained by high prevalence (99.3 %) of good practice among Ugandans.

Similar KAP studies among students, lectures, health workers, and rural market vendors in Uganda suggested that education level could play a key role in molding KAP in the community (11–13).

In our study occupations such as security agents, drivers, and business people had a low level of knowledge on COVID-19. These categories of the population need an urgent sensitization across the country to mitigate the spread of COVID-19. If measures are not considered, Uganda could continue to register increasing numbers of confirmed cases by these categories.

Our study was limited to participants who had smartphones, computers, tablets, and internet connectivity and had an understanding of English. Therefore, those with no smartphones and internet connectivity could not access the online form and participate in the survey. The survey captured the country's literate population, so it could not be generalized to the whole population. The knowledge and attitudes among uneducated people might be different from the findings of this survey.

Therefore, knowledge and attitudes toward COVID-19 of vulnerable populations deserve special research attention. There was an inadequate assessment of attitudes toward COVID-19, which should be developed via focus group discussion and in-depth interviews and constructed as multi-dimensional measures the same as for self-reported practice, which is not easy to evaluate as the survey was online. However, this was not possible due to the country's lockdown during the survey period, and one of the strategies observed by all population in the country was social distancing to avoid the spread of the COVID-19.

## Conclusion

At the time of the study, most Ugandans were knowledgeable, had a positive attitude, and observed good practices toward measures to prevent the spread of COVID-19 within the country. Despite these findings, there was lack of knowledge and attitude among specific populations, namely drivers, business entrepreneurs, and security personnel. These groups should be targeted for sensitization to avoid becoming the source of spread of the coronavirus disease. There is a dire need to mobilize all populations around the country to have the same level of knowledge, which will impact attitude and practice. The government of Uganda could use the health workers, teachers, and students to help in mobilization of all populations within the country about measures toward prevention of the spread of the coronavirus pandemic.

## Data Availability Statement

The original contributions presented in the study are included in the article/Supplementary Material, further inquiries can be directed to the corresponding author/s.

## Ethics Statement

Ethical clearance for the survey was obtained from the Institutional Research Ethical Committee of Kampala International University in Uganda (UG-REC-023/201914). As participants logged in online, a statement regarding the consent to participate in the survey was in the preamble of the questionnaire and could only proceed after reading the consent and accepting to participate in the survey. Participation in this survey was voluntary. Participants were free to withdraw from the survey at any time by not submitting their form online, and there was no repercussion.

## Author Contributions

RS and FS conceived and designed the survey, supervised the online data collection, and critically reviewed the manuscript. SM participated in conception of Google data form. LW and SN participated in online data collection. PK critically reviewed the manuscript. All authors contributed to the article and approved the submitted version.

## Conflict of Interest

The authors declare that the research was conducted in the absence of any commercial or financial relationships that could be construed as a potential conflict of interest.

## Footnotes

^1^https://docs.google.com/forms/d/18Ded-NFe65B6HnTFCwe4XzxBA-o3VfcJYogRjgcMsAA/edit
