# Supplementary material for: Knowledge, Attitude, and Self-Reported Practice Toward Measures for Prevention of the Spread of COVID-19 Among Ugandans: A Nationwide Online Cross-Sectional Survey
Source: Front Public Health. 2020 Dec 15;8:618731. doi: 10.3389/fpubh.2020.618731 (PMC7793670; doi:10.3389/fpubh.2020.618731)
Supplement: Supplementary file 1 [file Table_1.DOCX]

**Supporting information**

**S1 Table: Questionnaire: Knowledge, Attitude, self-reported practice and source of information items among participants**

| **Questions** | **Variables** | **Options** | **Choice** |
| --- | --- | --- | --- |
| 1. **Knowledge** | K1. The incubation period of Covid-19 is 2-14 days | 1. Yes 2. No |  |
|  | K2. What are the main modes of transmission of COVID-19?  (Multiple responses) | 1. Contact routes 2. Respiratory droplets 3. Airborne disease Disease from china 4. Witchcraft 5. Biological weapon 6. I don’t know |  |
|  | K3. Tick the signs or symptoms of Covid-19?  (Multiple responses) | 1. Dry Cough 2. Shortness of breath 3. Fever 4. sore throat 5. nose bleeding 6. Chest pain 7. I don’t know |  |
|  | K4. What are risks factors for severe illness of Covid-19?  (Multiple responses) | 1. Patient with underlying medical conditions 2. Elderly 3. Patients with immune suppression 4. Very young 5. Person who moves at night 6. None 7. I don’t know |  |
|  | K5. 20-30 seconds are required for washing hands with water and soap | 1. Yes 2. No |  |
|  | K6. Can infected individuals reduce the risk of spreading the virus to others by wearing face masks | 1. Yes 2. No |  |
|  | K7. Can uninfected people reduce the risk of infection by wearing facemasks when interacting with infected persons | 1. Yes 2. No |  |
|  | K8. Can widespread proper use of facemasks in a population facilitate the control of COVID-19 | 1. Yes 2. No |  |
|  | K9. Tick measures to observe for Community-Prevention?  (Multiple responses) | 1. Avoid public transport 2. Social distancing 3. Mass gatherings 4. School closure 5. Avoid market 6. go to church 7. I don’t know |  |
|  | K10. To eliminate all traces of the virus on your hands, what are the measures to take while washing hands?  (Multiple responses) | 1. Wet hands with running water 2. Apply enough soap to cover wet hands 3. Scrub all surfaces of the hands – including back of hands, between fingers and under nails – for at least 20 seconds. 4. Rinse thoroughly with running water 5. Dry hands with a clean cloth 6. I don’t know |  |
|  | K11. Tick instances when you should wash your hands?  (Multiple responses) | 1. Before and after eating 2. After touching money 3. After visiting a public space 4. After Coughing 5. After Sneezing 6. After blowing your nose 7. Before, during and after caring a normal person 8. Before, during and after caring a sick person 9. After touching surfaces outside of the home 10. I don’t know |  |
|  | K12. What are the treatment options of Coronavirus?  (Multiple responses) | 1. No available treatment 2. Follow medical advice 3. Follow prayers 4. Chloroquine 5. Fruits 6. warm water 7. Run and take traditional Herbs 8. I don’t know |  |
| 1. **Attitude** | A1. Do you worry about contracting COVID-19? | 1. Yes 2. No |  |
|  | A2. Do you follow recommendations given by the MOH or DHO to prevent the spread of COVID-19? | 1. Yes 2. No |  |
|  | A3. Do you agree that COVID-19 can be cured? | 1. Agree 2. Disagree |  |
| 1. **Practices** | P1. Tick measures to you are observing for Self-Prevention about COVID-19??  (Multiple responses) | 1. Self-monitoring 2. Use of Masks 3. Hand washing 4. Social distancing of more than two meters 5. Avoid gatherings 6. Stay at home 7. House Cleaning and Ventilation 8. Respiratory etiquette 9. social distance of less than one meter 10. I don’t know |  |
| 1. **Source of Information** | **What is your main source of information about COVID-19?**  **(One option)** | 1. Social Media |  |
|  |  | 1. Television |  |
|  |  | 1. Health Workers |  |
|  |  | 1. Radio |  |
|  |  | 1. Family and friends |  |
|  |  | 1. News Paper |  |
