# Supplementary material for: Knowledge, Attitude, and Self-Reported Practice Toward Measures for Prevention of the Spread of COVID-19 Among Ugandans: A Nationwide Online Cross-Sectional Survey
Source: Front Public Health. 2020 Dec 15;8:618731. doi: 10.3389/fpubh.2020.618731 (PMC7793670; doi:10.3389/fpubh.2020.618731)
Supplement: Supplementary file 2 [file Table_2.DOCX]

**S2 Table: Association of Knowledge level with Socio-demographic Characteristics**

| **Variable** | **Total (%)** | **Highly (%)** | **Moderate (%)** | **Poorly (%)** | **P-Value** |
| --- | --- | --- | --- | --- | --- |
| Sample size | 1763 (100) | 1411 (80) | 323 (18.3) | 29 (1.7) |  |
| Age group in years |  |  |  |  |  |
| 18 to 29 | 892 (100) | 737 (82.6) | 141 (15.8) | 14 (1.6) | 0.003 |
| 30 to 40 | 549 (100) | 436 (79.4) | 107 (19.5) | 6 (1.1) |  |
| 41 to 50 | 231 (100) | 178 (77.1) | 46 (19.9) | 7 (3) |  |
| 51 and above | 91 (100) | 60 (65.9) | 29 (31.9) | 2 (2.2) |  |
| Sex |  |  |  |  |  |
| Female | 759 (100) | 575 (75.8) | 163 (21.5) | 21 (2.8) | 0.001 |
| Male | 1004 (100) | 836 (83.3) | 160 (15.9) | 8 (0.8) |  |
| Marital status |  |  |  |  |  |
| Single | 891 (100) | 739 (82.9) | 141 (15.8) | 11 (1.2) | 0.003 |
| Married | 811 (100) | 630 (77.7) | 167 (20.6) | 14 (1.7) |  |
| Divorced | 42 (100) | 28 (66.7) | 11 (26.2) | 3 (7.1) |  |
| Others | 19 (100) | 14 (73.7) | 4 (21.1) | 1 (5.3) |  |
| Professional |  |  |  |  |  |
| Farmers | 247 (100) | 136 (55.1) | 95 (38.5) | 16 (6.5) | 0.001 |
| Business | 284 (100) | 196 (69) | 86 (30.3) | 2 (0.7) |  |
| Health workers | 418 (100) | 393 (94) | 25 (6) | 0 (0) |  |
| Household | 67 (100) | 45 (67.2) | 16 (23.9) | 6 (9) |  |
| Security | 49 (100) | 37 (75.5) | 11 (22.4) | 1 (2) |  |
| Student | 346 (100) | 301 (87) | 44 (12.7) | 1 (0.3) |  |
| Teacher | 119 (100) | 107 (89.9) | 12 (10.1) | 0 (0) |  |
| Driver | 50 (100) | 24 (48) | 23 (46) | 3 (6) |  |
| Others | 183 (100) | 172 (94) | 11 (6) | 0 (0) |  |
| Location (Region) |  |  |  |  |  |
| Western | 756 (100) | 583 (77.1) | 164 (21.7) | 9 (1.2) | 0.001 |
| Central | 517 (100) | 406 (78.5) | 95 (18.4) | 16 (3.1) |  |
| Eastern | 263 (100) | 227 (86.3) | 34 (12.9) | 2 (0.8) |  |
| Northern | 227 (100) | 195 (85.9) | 30 (13.2) | 2 (0.9) |  |

Other*: Widowed, Cohabiting, Separated and in relation

Other**: Technologist and Point of entry agent
